# Artificial intelligence for equitable remote patient monitoring in low-resource health systems

**DOI:** 10.1097/MS9.0000000000004193

**Published:** 2025-10-27

**Authors:** Md. Shafe Ul Alam, MD. Faisal Ahmed

**Affiliations:** Department of Health Sciences and Informatics, Bangladesh Institute of Innovative Health Research, Mirpur, Dhaka, Bangladesh

*To the Editor,*

This study strictly adheres to the Transparency in the reporting of Artificial INtelligence (TITAN) Guidelines and emphasizes that ‎other studies should follow this[1].

The recent surge in research exploring artificial intelligence (AI)-driven health care interventions marks a pivotal evolution in global medicine. Chronic diseases, particularly cardiovascular disease, hypertension, and chronic obstructive pulmonary disease, remain leading causes of mortality worldwide, collectively accounting for nearly 74% of all deaths^[2,3]^. Within this context, remote patient monitoring (RPM) has emerged as a transformative model for continuous, home-based disease management.

Evidence from high-income countries (HICs) demonstrates the feasibility, clinical utility, and economic value of RPM systems. A 2022 systematic review of economic evaluations confirmed that RPM is highly cost-effective for managing hypertension and heart failure, resulting in reduced hospital readmissions and improved adherence to therapy[4]. Similarly, a large US cohort study involving 6595 hypertensive patients reported a mean systolic blood pressure reduction of 7.3 mmHg following long-term RPM participation[5]. A United Kingdom meta-analysis further showed that telemonitoring achieved a 3.7 mmHg greater reduction in systolic blood pressure compared with usual care[6]. Collectively, these findings underscore that in well-resourced settings, RPM has matured into an integral, data-driven component of chronic disease management. In contrast, the situation in low- and middle-income countries (LMICs) is markedly different. Despite the escalating burden of chronic disease, RPM adoption remains largely confined to pilot-scale initiatives. For instance, an IoT-based remote monitoring prototype for asthma patients in Bangladesh demonstrated technical feasibility but lacked long-term outcome data or integration into clinical workflows[7]. Likewise, a systematic review of internet-based RPM systems in Asian LMICs highlighted potential benefits for disease control but revealed persistent barriers including inadequate digital infrastructure, limited interoperability, and low health literacy[8].

Several interrelated factors underlie this implementation gap:

- Inconsistent internet connectivity and unreliable power supply impede real-time data transmission.
- High device costs and the absence of reimbursement mechanisms hinder scalability.
- Limited digital literacy and insufficient clinician training restrict sustained utilization.
- Weak regulatory and data privacy frameworks constrain interoperability and multi-center collaboration.

We proposed research seeks to directly address these barriers by developing an AI-driven RPM framework specifically optimized for LMIC contexts. Leveraging machine learning algorithms such as Random Forest, Long Short-Term Memory, and Support Vector Machines, the model will analyze multimodal vital-sign data to enable predictive rather than reactive interventions. Distinct from existing HIC-centric systems, this framework will be designed for low-bandwidth environments and affordable wearable sensors, ensuring both scalability and equitable access. The novelty of this proposal lies in integrating AI with localized RPM systems to generate actionable predictions, reduce unnecessary hospital visits, and empower patients through real-time feedback loops. Beyond algorithmic innovation, it emphasizes contextual adaptation designing technologies attuned to the infrastructural and socioeconomic realities of LMICs. AI-enhanced RPM has the potential to redefine chronic disease management in resource-limited settings by bridging the gap between preventive care and real-time clinical insight. It represents not merely a technological advancement but a strategic opportunity to democratize digital health globally.

## Data Availability

No new data were generated or analyzed in this study. All supporting materials, datasets, and references are publicly available as cited in the article.
